# Sildenafil does not improve performance in 16.1 km cycle exercise time-trial in acute hypoxia

**DOI:** 10.1371/journal.pone.0210841

**Published:** 2019-01-17

**Authors:** Eric A. Carter, A. William Sheel, William K. Milsom, Michael S. Koehle

**Affiliations:** 1 School of Kinesiology, University of British Columbia, Vancouver, British Columbia, Canada; 2 Department of Zoology, University of British Columbia, Vancouver, British Columbia, Canada; 3 Division of Sports Medicine, University of British Columbia, Vancouver, British Columbia, Canada; University of Lausanne, SWITZERLAND

## Abstract

Sildenafil is a pulmonary vasodilator that has potential to mitigate the decrement in endurance performance caused by hypoxic pulmonary vasoconstriction. The purpose of this study was to determine the effects of sildenafil on pulmonary artery pressure, cardiac output, pulse oxygen saturation, and exercise performance at moderate simulated altitude. We hypothesized that sildenafil would reduce the decline in exercise performance in hypoxia by blunting the rise in pulmonary artery pressure and causing a relative increase in cardiac output and oxygen saturation. Twelve endurance trained men performed three experimental cycling trials at sea level and simulated moderate altitude of 3,000m (F_I_O_2_ = 0.147) after ingesting either a placebo or sildenafil 50 mg capsule in a double blinded fashion. Each test consisted of a warmup period, a 15-minute steady state period at 60% of peak power output, and a 16.1 km time-trial. All subjects experienced a decline in maximal exercise capacity in hypoxia that ranged from 6% to 24%. This decline was correlated with the reduction in pulse oxygen saturation in hypoxic maximal exercise. Sildenafil had no effect on pulmonary artery pressure, cardiac output, or pulse oxygen saturation measured during steady state exercise. There was no effect of sildenafil on mean power output during the time-trial. During high intensity cycle exercise in acute, moderate hypoxia pulmonary artery pressure is unaffected by sildenafil and does not appear to influence cardiovascular function or exercise performance.

## Introduction

Athletic competitions are frequently held at moderate altitude (1500–2999 metres, equivalent to a barometric pressure (P_B_) of 635–525 mmHg) for example: the 1968 Mexico City Olympics at 2250 meters, the 2017 Ski Mountaineering World Championships in Piancavello, Italy at 2251 meters, or the Livigno Sky Running World Cup at 3005 meters. Such an altitude, while not so high as to cause altitude-related illness, can negatively impact endurance performance for both athletes in competition and recreationally active individuals travelling to altitude. Endurance performance is impaired by the decrease in ambient partial pressure of oxygen (PO_2_), leading to reduced oxygen delivery to muscle tissues [1].

In an effort to maximize oxygen delivery to exercising muscle, hypoxemia results in smooth muscle relaxation in the systemic circulation and smooth muscle contraction in the pulmonary vasculature. This contraction, termed hypoxic pulmonary vasoconstriction (HPV), is modulated by reduced arterial PO_2_ and occurs within seconds [2,3]. In healthy humans at altitude, the effect of the HPV response is to divert blood flow from lung units that are less well ventilated (and hence have a low PO_2_) towards better ventilated units, increasing arterial oxygen content (C_a_O_2_) [3].

The result of the HPV response in acute, global hypoxia (for example, at high altitude) is a marked increase in pulmonary blood pressure, usually measured in the pulmonary artery during systole (PAP). Increases in PAP at altitude are further compounded during exercise, causing pressures to more than double [4]. Elevated PAP increases right ventricular afterload, significantly reducing stroke volume (SV) and thereby cardiac output (CO). When CaO_2_ is reduced in hypoxia, oxygen uptake (V̇O_2_) is maintained by increasing CO. However, if CO is already at maximal levels, V̇O_2max_ and performance are limited [5]. During submaximal exercise, when CaO_2_ is reduced, ventilation (V̇_E_), heart rate (HR), contractility, and CO are increased to maximize O_2_ delivery at a given intensity and partially offset the reduction in oxygenation of arterial blood [4].

Athletes use a variety of methods to minimize the detrimental effects of hypoxia on athletic performance with varying degrees of success. One strategy is to lower PAP by pharmaceutically promoting pulmonary smooth muscle relaxation, thereby increasing SV and CO, with the intended result of improving performance. Sildenafil is a phosphodiesterase-5 (PDE-5) inhibitor that competes with cyclic guanosine 3’, 5’ monophosphate (cGMP) for binding sites on PDE-5, preventing cGMP breakdown and increasing the concentration available for smooth muscle relaxation [6]. Sildenafil does not increase smooth muscle relaxation by increasing nitric oxide (NO) availability, but instead by inhibiting the mechanism that reduces the effects of NO.

Previous research has been inconclusive on the effectiveness of sildenafil to reduce HPV and improve exercise performance during acute moderate- and high-altitude exposure. Several studies have shown a strong effect of sildenafil in reducing PAP at rest [7–9] and during exercise [7,9,10]. However, only two [7,11] of the five studies that measured CO [7,10–13] showed an increase in PAP because of sildenafil use during exercise in hypoxia. Only three [7,10,11] of six studies that measured performance outcomes [7,8,10–13] showed an increase with sildenafil use.

The purpose of this study was to investigate the use of sildenafil to improve exercise performance in hypoxia by comparing changes in PAP with the decrement in exercise performance. We hypothesized that during exercise in acute moderate hypoxia (F_I_O_2_ = 0.147), 50 mg sildenafil would significantly reduce PAP and increase CO, resulting in improved exercise performance. We also expected that a greater decline in hypoxic exercise performance would be correlated with a higher PAP during steady state exercise.

## Materials and methods

Twelve male competitive cyclists participated in this study. Their age, height, and body weight, [mean ± standard deviation (SD)] were 37 ± 9 years, 180 ± 4 cm, and 71.1 ± 8.1 kg. Participants’ mean V̇O_2max_ was 68.6 ± 8.0 mL kg^-1^ min^-1^. All participants were sea-level residents who had not travelled above 2,000m (excluding commercial air travel) in the preceding six months. While this altitude threshold is higher than might be ideal and possibly considered a limitation, all participants reside at, and should be considered, acclimatized to sea-level. Participants maintained a consistent amount of endurance training (five sessions per week for more than one year) and competed regularly in endurance cycling or triathlon competitions (≥3 competitions per year). The study was approved by the Clinical Research Ethics Board at the University of British Columbia and all procedures were in accordance with the ethical standards of the 1964 Helsinki Declaration. Participants were informed of the procedures and possible risks involved in the study and informed consent was obtained.

**Fig 1:**
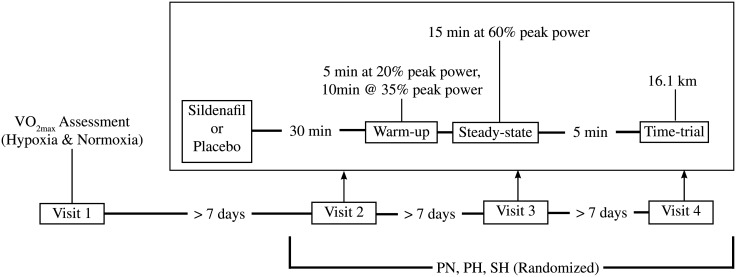
Study timeline. PN, placebo normoxic test; PH, placebo hypoxic test; SH, Sildenafil hypoxic test.

Participants visited the Environmental Physiology Laboratory on four occasions (Fig 1). During the first visit, participants completed two maximal exercise tests on a magnetically braked cycle ergometer (Racermate, South Dakota, USA) under a normoxic (NOR; F_I_O_2_ = 0.209) and normobaric hypoxic condition (HYP; F_I_O_2_ = 0.147, equivalent to a simulated altitude of 3,000m). The HYP condition was produced using a custom hypoxic enclosure (Colorado Altitude Training, Colorado, USA) that reduces the O_2_ concentration of room air (normobaric hypoxia (NH)) and delivers it to the participant using large bore tubing and one-way valve breathing mask. The order of these tests was randomized. Workload started at 100 watts and increased in a ramp fashion, 1 watt every 2 seconds until exhaustion (an inability to maintain the prescribed cadence (>60 rpm)). During the test procedure, respiratory gases were collected and analyzed using fast response O_2_ and CO_2_ gas analyzers and pneumotachometer (Vacumed, California, USA). Pulse oxygen saturation (S_P_O_2_) and HR were collected with an ear-clip using a Nonin 9600 Pulse Oximeter (Nonin Medical, Minnesota, USA). The signal from these devices was stored digitally on a PowerLab data acquisition system (ADInstruments, Dunedin, New Zealand) with a mean value calculated every 10 seconds.

During the three remaining visits, participants completed experimental trials in NOR with a placebo or HYP with either placebo or the study drug, sildenafil, using a randomized crossover design. Sildenafil was administered as a single oral dose of 50 mg, the dose used most commonly in studies of exercise performance in hypoxia and previously shown to elicit a decrease in PAP [7]. Participants were blinded to the condition and drug. The placebo and sildenafil were compounded locally (McDonalds Pharmacy, British Columbia, Canada) to be indistinguishable. NOR and HYP sessions were completed a minimum of 7 days after maximal exercise testing and with 7 days between each session.

Each of the three experimental visits began with participants ingesting a placebo or sildenafil capsule (randomized) with a glass of water (150 mL). Approximately 30 minutes after ingestion, participants began the warmup protocol. The warmup involved 5 minutes at 20% of peak power (determined during maximal exercise test) followed by 10 minutes at 35% peak power. At 44 ± 2 min, the steady state portion of the trial began. During the NOR steady state, subjects rode at 60% of peak power as measured during the NOR maximal exercise test (233 ± 18 watts). During HYP steady state, subjects rode at 60% of peak power as measured during the HYP maximal exercise test (197 ± 14 watts). The pulmonary vasodilatory effects of sildenafil during steady state exercise were estimated using echocardiography. A certified ultrasound technician measured the aortic root diameter and aortic velocity time integral (VTI) as well as the velocity of the regurgitant tricuspid (VTR) jet using a Vivid I ultrasound and 3S cardiac probe (General Electric, Massachusetts, USA). Stroke volume and PAP were calculated using Eqs 1 and 2. Right atrial pressure (RAP) was estimated to be 5 mmHg in all participants. Measurements were taken between 10 and 15 minutes of steady state exercise time. After 15 min of steady state exercise, resistance was reduced to 50 watts for 5 minutes. After 103 ± 4 minutes, participants began the time-trial (TT) portion of the visit. Participants were instructed to complete a flat, 10-mile (16.1 km) simulated cycling course as quickly as possible. Participants were able to see their current cadence and distance completed but no other performance or physiologic variables. Order of visit was not found to have any effect on time-trial performance (*p* > 0.05).

$$\text{SV}=\left(3.145*{\left(\frac{\text{Aortic}\;\text{Root}}{2}\right)}^{2}\right)*\text{VTI}$$ (1)

$$PAP\;=\;{\left(4*VTR\right)}^{2}+RAP$$ (2)

The hypoxic dose for each visit was calculated as P_I_O_2_ [14] using the P_B_ at the time of the visit and F_I_O_2_ and is shown in Table 1. There was no difference in P_I_O_2_ between hypoxic conditions (*p* > 0.05).

**Table 1:** Effects of sildenafil during time-trial exercise.

Variable	*Placebo NOR (PN)*	*Placebo HYP (PH)*	*Sildenafil HYP (SH)*
Power mean (watts)	275 ± 26*	230 ± 22	230 ± 20
Power mean (watts kg^-1^)	3.9 ± 0.6*	3.3 ± 0.5	3.3 ± 0.5
HR mean	172 ± 10	168 ± 8	164 ± 17
HR max	183 ± 11*	177 ± 9	175 ± 9
V̇O_2avg_ (ml kg^-1^ min^-1^)	46.0 ± 6.2*	37.9 ± 5.0	36.5 ± 5.7
Barometric Pressure (mmHg)	748 ± 5	748 ± 7	752 ± 7
P_I_O_2_ (mmHg)	144.9 ± 1.8*	103.1 ± 1.0	103.6 ± 0.9

HR, heart rate; V̇O_2_, oxygen uptake; S_P_O_2_, pulse oxygen saturation; P_I_O_2_, partial pressure of inspired oxygen.

* Normoxic values significantly different from PH and SH (*p* < 0.05).

### Statistical analysis

All data are presented as mean ± SD. A two-way analysis of variance (ANOVA) with repeated measures was used to confirm the interaction (F_I_O_2_ x drug). When the ANOVA revealed a significant effect, a post-hoc Tukey-Kramer test was performed to identify differences. Pearson’s correlation coefficient (*r*) was used as a measure of linear correlation between descriptive variables included in the study and exercise performance.*p* values < 0.05 were considered statistically significant.

## Results

**Fig 2:**
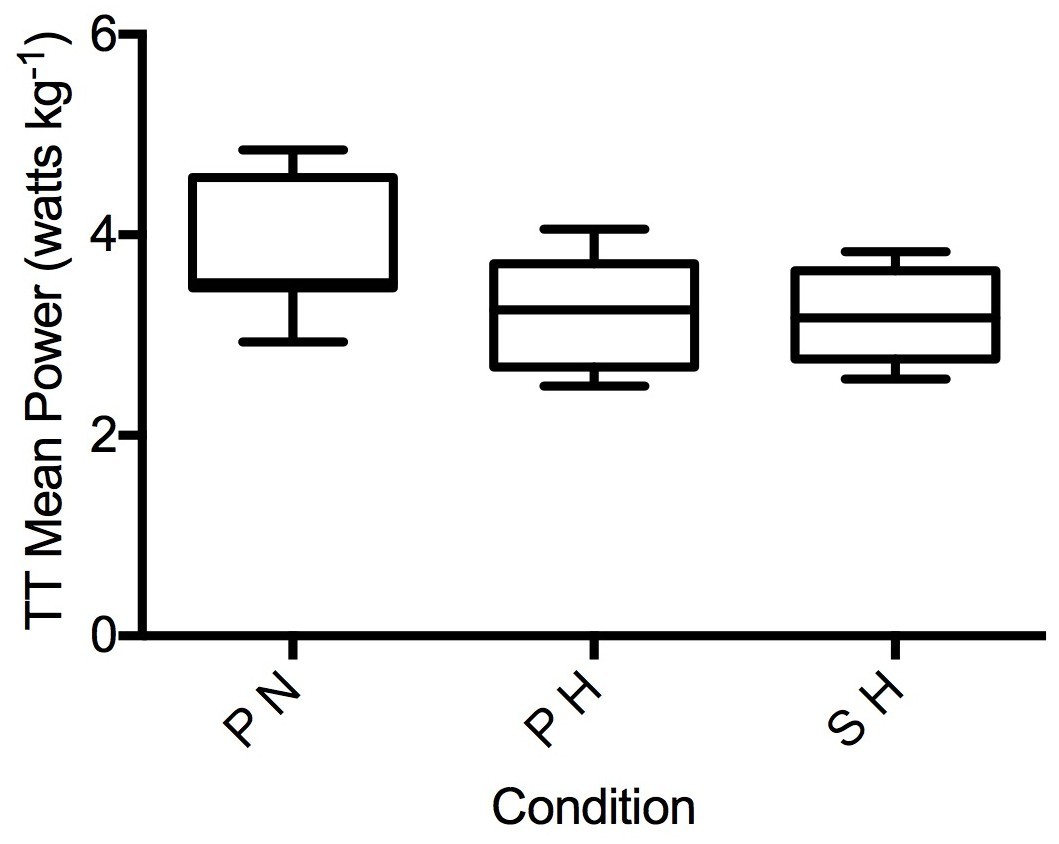
Relative mean power output during time-trial. PN, placebo in normoxia; PH, placebo in hypoxia; SH, sildenafil in hypoxia; *Indicates normoxic values significantly different from PH and SH (*p* < 0.05).

In 11 participants (one was unable to complete the trial due to fatigue), normobaric hypoxia significantly reduced time-trial performance (normalized to mean power output per kilogram of bodyweight) in both the placebo hypoxia (PH) and sildenafil hypoxia (SH) condition compared to normoxia (main effect for F_I_O_2_: *p* < 0.01) (Fig 2). There was no significant difference between the PH and SH trials (*p* > 0.05).

**Fig 3:**
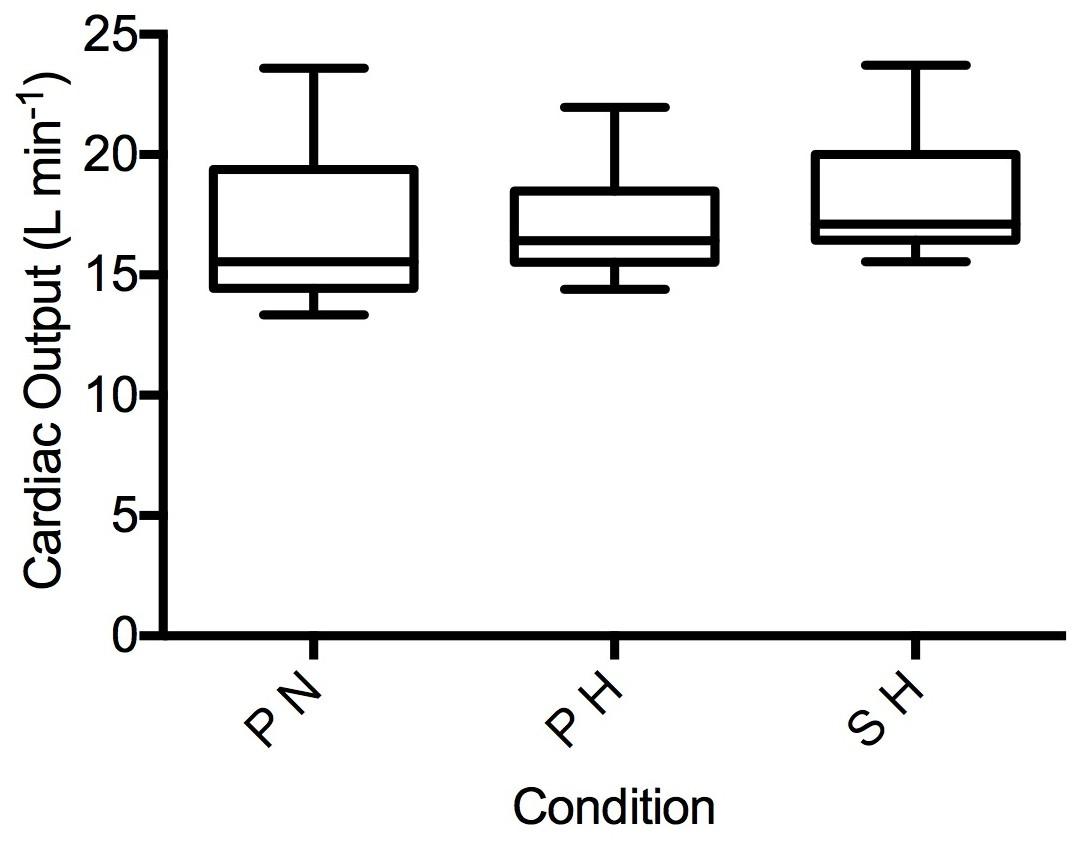
Cardiac output during time-trial. PN, placebo in normoxia; PH, placebo in hypoxia; SH, sildenafil in hypoxia.

**Fig 4:**
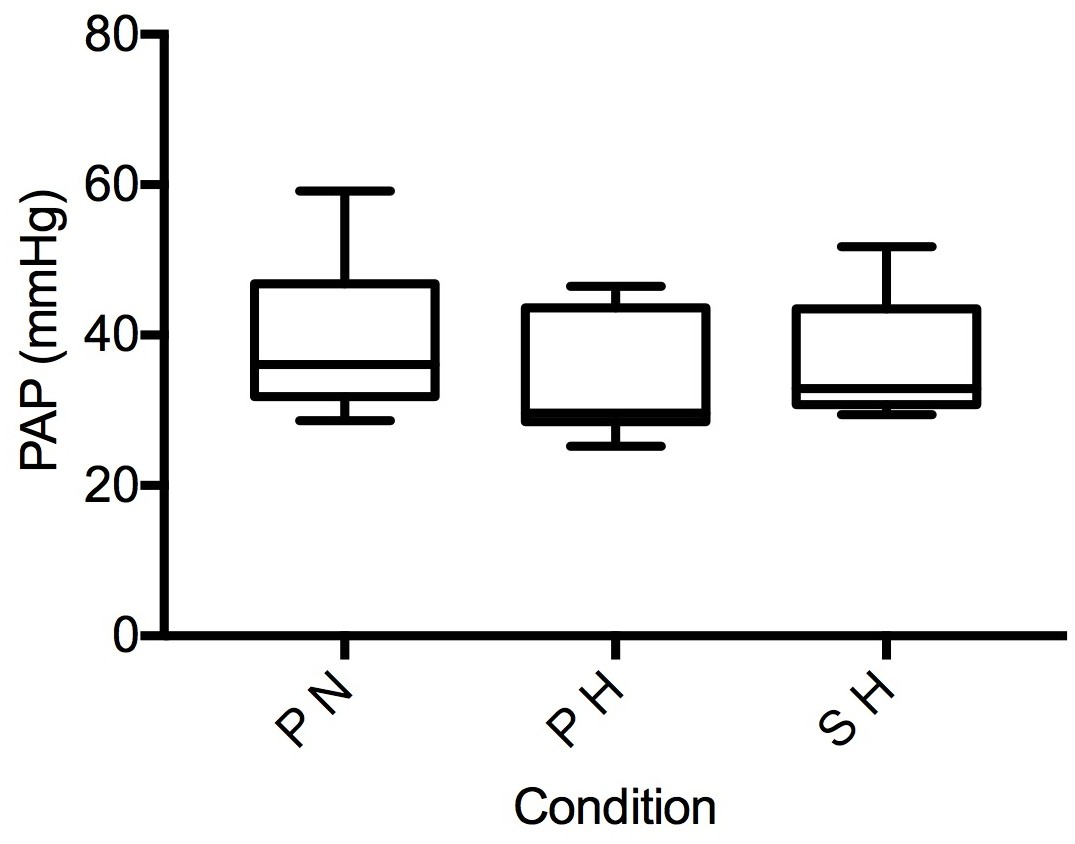
Pulmonary artery pressure during time-trial. PAP, pulmonary artery pressure in systole; PN, placebo in normoxia; PH, placebo in hypoxia; SH, sildenafil in hypoxia.

There was no significant difference between the three experimental conditions (*p* > 0.05) in CO in 10 participants with measurable aortic VTI during steady state exercise (Fig 3). Similarly, there was no significant difference in PAP between conditions (*p* > 0.05) in six participants with a measurable TR jet (Fig 4). Additional steady state exercise data is found in Table 2. The individuals with a detectable TR jet did not differ in CO or performance from those in whom a jet was not detected (*p* > 0.05).

**Table 2:** Effects of sildenafil during steady state.

*Variable*	*n =*	*Placebo Normoxia (PN)*	*Placebo HYP (PH)*	*Sildenafil HYP (SH)*
Power (watts)	12	233 ± 17*	197 ± 14	197 ± 4
Power (watts kg^-1^)	12	3.3 ± 0.3*	2.8 ± 0.3	2.8 ± 0.3
V̇O_2_ (ml kg^-1^ min^-1^)	12	42.7 ± 5.4*	34.6 ± 3.6	32.9 ± 5.7
Cardiac Output (L min^-1^)	10	16.8 ± 3.2	17.1 ± 2.4	18.3 ± 2.6
Stroke Volume (mL)	10	110.4 ± 22.6	112.2 ± 13.4	115.2 ± 15.8
Systolic Pulmonary Artery Pressure (mm Hg)^X^	6	39.3 ± 10.9	33.9 ± 8.6	36.5 ± 8.5
S_P_O_2_ (%)	12	96 ± 4*	83 ± 4	81 ± 7
Systolic Blood Pressure (mmHg)^a^	12	117 ± 11	121 ± 12	122 ± 10
Diastolic Blood Pressure (mmHg)^a^	12	72 ± 5	74 ± 9	73 ± 7
Mean Arterial Pressure (mmHg)^a^	12	89 ± 7	90 ± 9	87 ± 6

V̇O_2_, oxygen uptake; S_P_O_2_, pulse oxygen saturation; HYP, Hypoxia.

^a^Measured at rest prior to exercise bout.

*Hypoxic values (PH and SH) significantly different from normoxia (*p* < 0.05).

^X^Insufficient data for statistical comparison.

**Fig 5:**
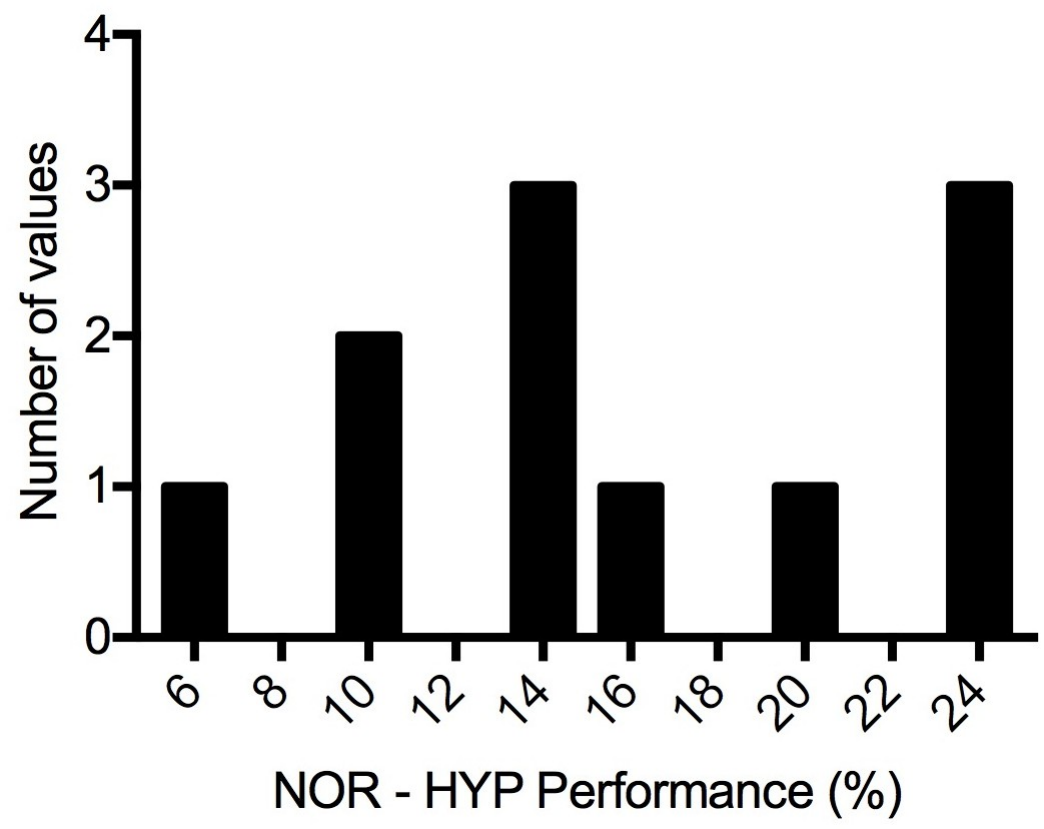
Range of decrement in time-trial performance from normoxia to hypoxia. NOR, normoxic maximal exercise test; HYP, hypoxic maximal exercise test.

While hypoxia reduced the exercise capacity of all participants, some were affected more than others. Fig 5 shows the range of the decrement in TT performance between condition placebo normoxia (PN) and PH from 6% to 24%. Participants who exhibited the greatest decrement in performance in HYP did not see any added benefit from sildenafil compared to those who had a minimal decrement in performance (*r* = 0.23, *p* > 0.05). No other variables measured in the experiment were correlated with the decrement in TT performance from NOR to HYP (*p* > 0.05).

**Fig 6:**
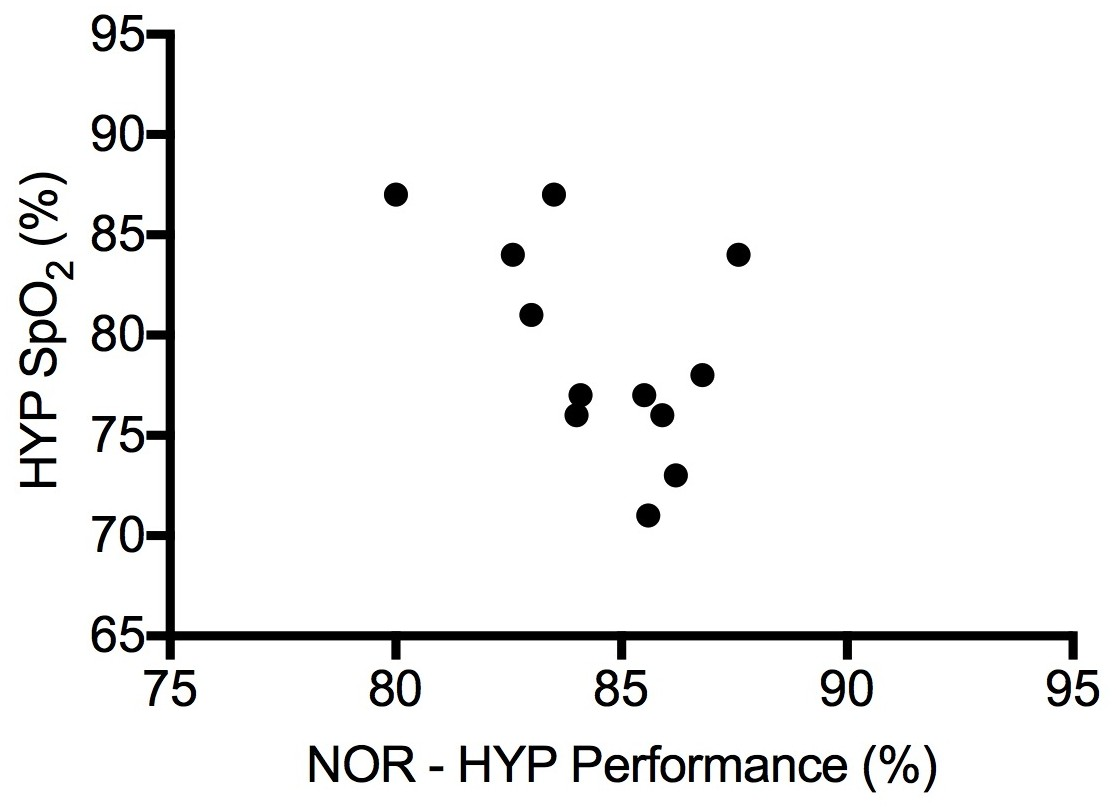
Oxygen saturation in hypoxic maximal exercise test predicts performance decrement in hypoxia. S_P_O_2_, pulse oxygen saturation; NOR, normoxic maximal exercise test; HYP, hypoxic maximal exercise test.

Six participants demonstrated signs of exercise-induced arterial hypoxemia (EIAH) during the NOR maximal exercise test (S_P_O_2_ < 95%). Minimum S_P_O_2_ during maximal exercise dropped significantly from NOR to HYP (Table 3). During steady state exercise, S_P_O_2_ dropped significantly from PN to PH and SH (Table 2). Fig 6 shows a negative correlation in the decrease in performance (peak power) between hypoxic and normoxic maximal exercise tests and S_P_O_2_ measured in the hypoxic maximal exercise test (*r* = -0.78, *p* < 0.01). During maximal exercise in both normoxia and hypoxia, V̇_E_/V̇O_2_ did not correlate with S_p_O_2_ (*r* = -0.38, *p* > 0.05) and a t-test of subjects with high and low S_p_O_2_ revealed no difference (*t = 1*.*46*, *p* > 0.05).

**Table 3:** Effects of hypoxia on maximal exercise test.

*Variable*	*NOR*	*HYP*
Power peak (watts)	389 ± 29	329 ± 24*
Power peak (watts kg^-1^)	5.5 ± 0.6	4.7 ± 0.5*
V̇O_2max_ (ml kg^-1^ min^-1^)	68.65 ± 8.03	53.37 ± 6.72*
HR max (bpm)	184 ± 10	182 ± 10*
S_P_O_2_ (%)	92.8 ± 5.2	79.2 ± 5.3*
V̇_E_/V̇O_2_	37.58 ± 5.42	48.28 ± 4.32*

HR, heart rate; V̇_E_, ventilation; V̇O_2_, oxygen uptake; S_P_O_2_, pulse oxygen saturation;

*Hypoxic values significantly different from normoxia (*p* < 0.05).

## Discussion

The primary findings of this study were that sildenafil did not improve exercise performance during a 16.1 km cycle TT at a simulated moderate altitude of 3,000 m and resulted in no change in CO or PAP during sub-maximal exercise indicating that sildenafil is unlikely to be useful to athletes performing at these altitudes. There was a wide range in the detrimental effect of hypoxia on exercise performance in the TT and during the maximal exercise test. The range in the decrement in performance in the maximal exercise test was not correlated with PAP but instead driven primarily by variation in S_P_O_2_ in hypoxia.

### Effect of sildenafil on performance

Our results showing a lack of effect of sildenafil on endurance performance in hypoxia are in line with the results by three of the six studies identified in the introduction [8,12,13], all three of which used the same dose of sildenafil and performance metrics that included a maximal exercise test, a 6 km time-trial, and a 15 km time-trial respectively. This may be the result of a varying aerobic component in the performance outcome or a threshold altitude based on variation in the equivalent level of hypoxia (ranging from 3000 m to 5500 m). Interestingly, the results by Ghofrani, *et al*., showed sildenafil significantly improved performance in a maximal exercise test at 5500 m despite no other noticeable difference in protocol [7].

### Effect of sildenafil on PAP

In our sample of twelve participants, only six had a measurable TR jet, required to estimate PAP, due to the nature of echocardiography-based measures. This limited our statistical analysis, however of those participants with a TR jet, the mean PAP with sildenafil was 2.6 ± 15.1 mmHg lower than placebo suggesting that if there were a difference in PAP between the two treatments, it would be small and highly variable between-individuals. Because of the small sample size, PAP results are insufficient to draw any conclusions and should only be used to determine trends. Previous research has shown a low prevalence of TR jet in healthy sedentary humans (15–18%) but higher rates in trained athletes (76%) [15,16]. Future research seeking to use these estimations should anticipate a lower rate of measurable TR jets or utilize a different technique to measure PAP.

We hypothesized an increase in CO with sildenafil due to a relative decrease in PAP over placebo but observed that CO was not significantly affected by sildenafil. While this does not support our hypothesis, it supports the lack of observed effect of sildenafil on PAP. When subjects were grouped into those with and without a measurable TR jet, there were no differences in CO or performance, suggesting that if CO and performance are mediated by changes in PAP, the effect of sildenafil on PAP in the individuals without a TR jet was likely similarly unchanged. This finding also suggests that the presence of TR jets alone did not affect performance. While the low number of PAP measurements was a limitation within the study, our data indicate that PAP was not significantly reduced by sildenafil, which is supported by CO and performance data.

The lack of change in PAP is difficult to explain. To minimize the chance that it was caused by medication timing or dose, exercise tasks were timed to occur during estimated peak plasma concentration levels of sildenafil (~1 hr after ingestion) and well within the 4-hour half-life [17]. The dose was chosen because of the very low inhibitory concentration of sildenafil, such that with the 50 mg dose used in this study, PDE-5 would be 86% inhibited [18]. Previous research has successfully shown a reduction in PAP in hypoxia using a 50 mg dose [7,8,11,13]. This is the most commonly used dose in the literature reviewed and it is unlikely that adjusting the timing or dose of sildenafil would have a significant impact on the outcome of the study though supra-therapeutic doses may be used by athletes striving for a performance advantage and this may account for research that suggests a possibility of performance improvement [10].

We believe that the most likely explanation for the lack of effect of sildenafil is twofold. First, the increase in PAP is the result of multiple processes that cannot be entirely controlled with a PDE-5 inhibitor. The sensor that drives the HPV response is believed to reside in the mitochondria of the pulmonary artery smooth muscle cells and relies on reduction-oxidation chemistry for calcium and potassium channel activation [19]. While the NO pathway acts as an effector on HPV, it may be secondary to direct O_2_ sensing in the mitochondria.

Second, the vasodilatory effect of sildenafil appears to be small compared to the large increases in PAP because of exercise and hypoxia in healthy athletes. Studies in subjects at rest have shown sildenafil to have a very small to negligible effect on PAP. Guazzi *et*. *al*. showed that sildenafil did not change resting PAP at all in healthy subjects [20]. During moderate intensity cycle exercise, PAP can more than triple, from 12.2 mmHg at rest to 37.2mmHg [4]. If the magnitude of the reduction in PAP with sildenafil does not increase proportionately to the increase in PAP during hypoxic exercise, then it’s possible that the vasodilatory effect of sildenafil is not of sufficient magnitude to overcome the vasoconstrictive effect of exercise in hypoxia.

Our findings indicate that sildenafil is an insufficient pulmonary vasodilator to overcome the combined effects of HPV and exercise in hypoxia and suggests that increases in PAP because of HPV may not be the sole factor for the decrement in performance in hypoxia.

### Range in hypoxic decrement

All subjects experienced a hypoxia-related decline in TT performance however, the range in these declines was greater than expected. Some athletes saw a minimal decrement in performance in hypoxia (6% decrease in TT power compared to normoxia) while others performed much worse (24% decrease compared to normoxia). For two athletes with similar fitness at sea-level but competing at altitude, this would indicate a dramatic difference in race result. We should note, there is some debate over the difference in the physiologic effect of NH vs. hypobaric hypoxia (HH). The higher V̇_E_ observed in NH [21] indicates that O_2_ delivery could be higher compared to an equivalent altitude in HH, possibly causing us to underestimate the decrement in performance observed in this study.

The reduction in V̇O_2max_ in hypoxia was also variable. Previous studies have shown athletes with a higher sea-level V̇O_2max_ have the largest decrement in hypoxic V̇O_2max_ [22]. The mean decrement in V̇O_2max_ was 15.3 mL kg^-1^ min^-1^ and sea-level maximal exercise test peak wattage dropped between 12% and 20% in the hypoxic test. This decrement was significantly smaller than expected, based on predictive equations [23], possibly because the mean baseline V̇O_2max_ in our study was higher than most of the studies included in MacInnis’ meta-analysis. Contrary to several previous studies [1,24,25], we did not find any evidence in our results that S_P_O_2_ or EIAH status during sea-level maximal exercise would correlate with the decrease in V̇O_2max_ in hypoxia. This may suggest that participants in this study showed a greater tolerance for hypoxia compared to those included in the meta-analysis. A greater hypoxic tolerance could also be a contributing factor to the lack of effect that sildenafil had on our outcome measures.

The decrement in performance (in either the TT or maximal exercise test) between normoxia and hypoxia was not correlated with PAP or CO. There was no correlation between age or fitness (as measured by V̇O_2max_, performance in the TT, or maximal exercise test) and the decrement in performance. We did however, find a relationship showing the minimum S_P_O_2_ during the hypoxic maximal exercise test to be a predictor of the difference in peak power between NOR and HYP maximal exercise tests. Those individuals with the lowest S_P_O_2_ during peak exercise in hypoxia had the largest drop in peak power between their normoxic and hypoxic tests. It’s important to note that this decrement did not predict the difference in hypoxic TT performance, a finding that we will discuss in the next section.

### Arterial oxygen saturation is the major determinant of hypoxic V̇O_2max_

The correlation between the variation in the reduction in arterial O_2_ saturation (S_a_O_2_) and the variation in the decrement in maximal performance in hypoxia (specifically V̇O_2max_), has been previously reported, most notably in highly fit athletes [1]. Several explanations exist, although most conclude that the mechanism is likely multifactorial. Classically, the variation in hypoxemia can be attributed to some combination of: hypoventilation, diffusion limitation, and V̇/Q̇ mismatch [17].

The question remains however, why did the drop in S_P_O_2_ predict the decrement in performance in the maximal exercise test but not in the TT? The differences in V̇O_2_ between the two tests indicate significantly different energy systems in use. During the maximal exercise test, athletes reached the limit of their aerobic capacity (well above the anaerobic threshold) while during the TT, the athletes maintained a sub-maximal output at ~68% of V̇O_2max_. If we consider the factors affecting variation in S_a_O_2_ introduced above (inadequate ventilation, diffusion limitation, and V̇/Q̇ mismatch) we see why we might expect differences in S_a_O_2_ between maximal and sub-maximal exercise.

During submaximal exercise, a rise in V_E_ (part of the hypoxic ventilatory response or HVR) would be expected to help mitigate the loss in S_a_O_2_, while during maximal exercise, evidence suggests that some highly-trained athletes may experience a degree of mechanical limitation to expiratory flow preventing a rise in V_E_ that would at least partially defend S_a_O_2_ [26,27]. During submaximal exercise, SV and HR would have the capacity to increase CO to maintain S_a_O_2_, while during maximal exercise, SV and HR would be unable to increase beyond peak values. Maximal levels of CO can circulate blood through the pulmonary capillaries much faster than at rest, reducing the capillary transit time to less than that required to completely saturate the passing red blood cells [1,28] and increased pressure can damage the capillary-alveolar membrane resulting in extra-capillary fluid, further impairing gas exchange through diffusion limitation [17,29]. At lower exercise intensity, most of the CO passes through the gas exchange zones of the lungs. During maximal exercise [30], compounded by hypoxia [31], the opening of small blood vessels called intra-pulmonary arteriovenous anastomoses allows blood to bypass the air-exchange zone of the lung. Blood traversing these vessels mixes with pulmonary venous blood returning to the left heart, reducing overall S_a_O_2_ and possibly impacting PAP as well. Finally, during maximal exercise, we expect S_a_O_2_ to be lower due to a right shifted oxyhemoglobin dissociation curve that reduces the affinity for O_2_ loading at the lungs [29] and the ability to adopt a pacing strategy that allows the athlete to achieve unsustainable workloads.

All the factors discussed above that implicate S_a_O_2_ as the primary determinant of maximal exercise performance in hypoxia also favor maintenance of S_a_O_2_ sub-maximally. This supports our findings that suggest S_P_O_2_ is not a good indicator of sub-maximal TT performance in hypoxia.

## Conclusion

This single-blind, randomized controlled trial confirmed that 50mg of sildenafil does not improve exercise performance during a 16.1 km cycling time-trial at a simulated altitude of 3, 000 m. Furthermore, exposure to hypoxia negatively affects the endurance performance of athletes differently and in a wide range, likely mediated by variation in S_a_O_2_. Sea-level fitness is unrelated to this range in performance and increasing fitness does not protect against the performance decline at moderate altitude. These findings indicate that the use of 50mg sildenafil is not ergogenic in moderate hypoxia. Currently, sildenafil is not a prohibited or monitored substance as per the World Anti-Doping Agency. Our results indicate that the policy need not be changed.

## Supporting information

S1 Dataset A complete dataset from this experiment can be found in the supplementary information. (XLSX)
